# Supplementary material for: Viral gene drive in herpesviruses
Source: Nat Commun. 2020 Sep 28;11:4884. doi: 10.1038/s41467-020-18678-0 (PMC7522973; doi:10.1038/s41467-020-18678-0)
Supplement: Supplementary file 1 — Supplementary Information [file 41467_2020_18678_MOESM1_ESM.pdf]

Primer name	Sequence	
UL23 left homology arm F	GCGACGACGATCGTTTCTTT	Fig. 3d, S1b
UL23 left homology arm R	CTTGTAGTCTCCGTCGTGGT	Fig. 3d, S1b
UL23 Right homology arm F	CAACTTGAAAAAGTGGCACCGA	Fig. 3d, S1b
UL23 Right homology arm R	TTGTGAACGCGGTTATCGTG	Fig. 3d, S1b
UL23 cut site F	GCTTGGGGCATAAAACACCG	Fig. S1b
UL23 cut site R	CCCAGGTACAGTTCAGACGG	Fig. S1b
eGFP F	CTTCAGCTCGATGCGGTTCA	Fig. 3b-c
eGFP F	GACGTAAACGGCCACAAGTTC	Fig. 3b-c
mCherry F	AGTTCATGCGCTTCAAGGTG	Fig. 3b-c
mCherry F	GTCTTGACCTCAGCGTCGTA	Fig. 3b-c
Genotyping TB40/E mCherry F	TCCCGTACTGCATGTTCCAC	Fig. S1c
Genotyping TB40/E mCherry R	CAGAGAACGTCACACCGTCA	Fig. S1c

Supplementary Table 1: Primers

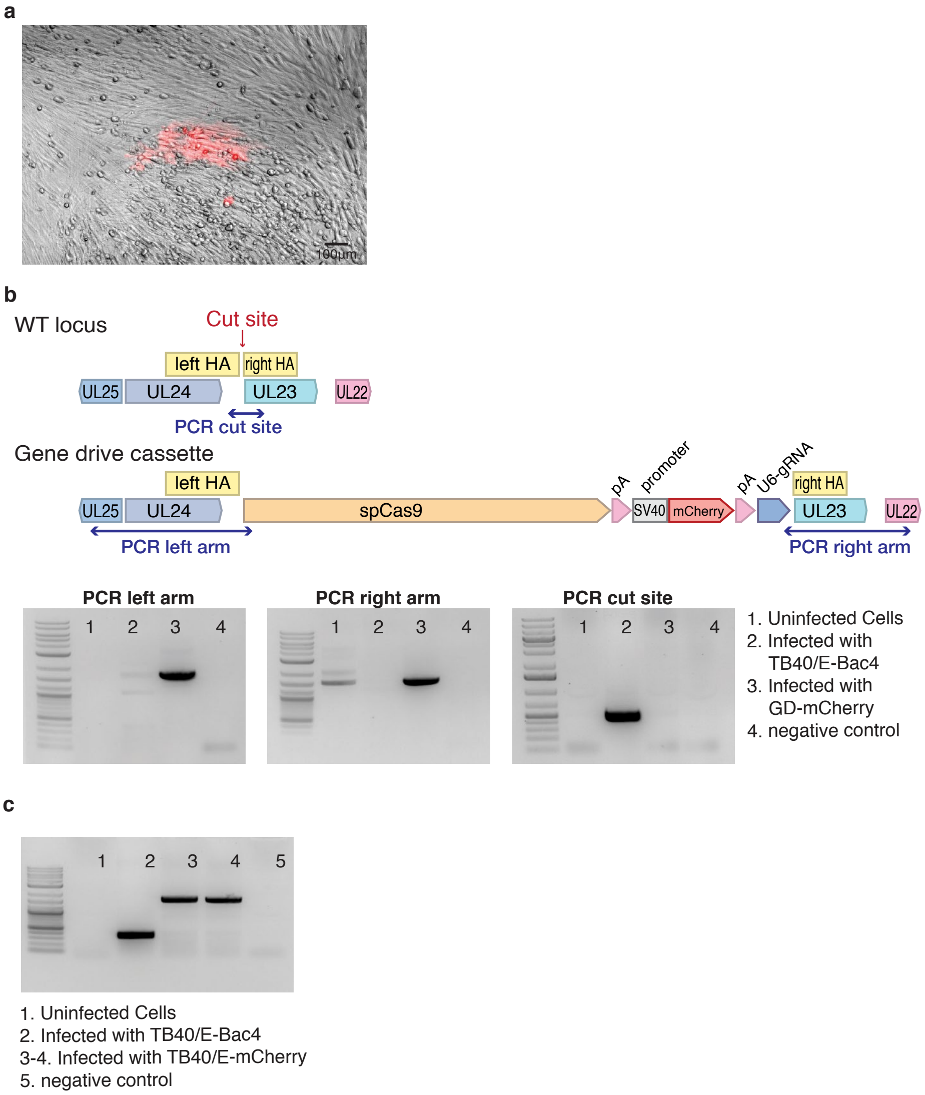

Supplementary Figure 1: Generation of gene drive viruses.
a, Representative image of a mCherry-expressing viral plaque spreading into human fibroblasts, approximately 10 days after transfection with the gene drive donor plasmid and infection with hCMV (TB40/E strain). **b**, Genotyping PCR of pure GD-mCherry population and primer localization. **c**, Genotyping PCR of pure TB40/E-mCherry population.
Source data are provided as a Source Data file.

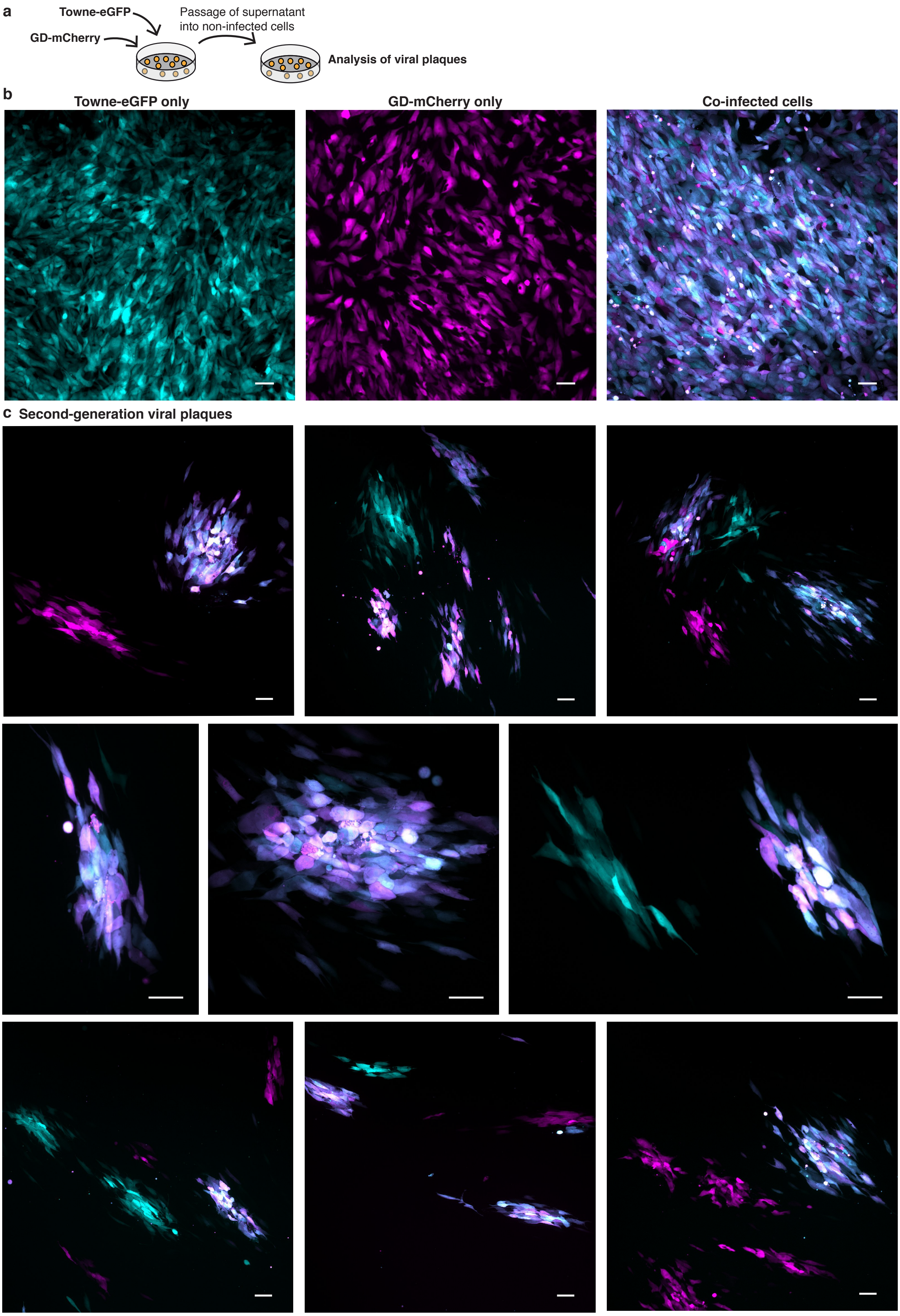

Supplementary Figure. 2: Recombinant viral plaques.
a, Experimental scheme: fibroblasts were coinfected with Towne-eGFP and GD-mCherry, and supernatants used to infect fresh cells. **b**, Images of cells infected with Towne-eGFP, GD-mCherry, or both. **c**, Images of second generation recombinant viral plaques.
Scale bars: 100µm.

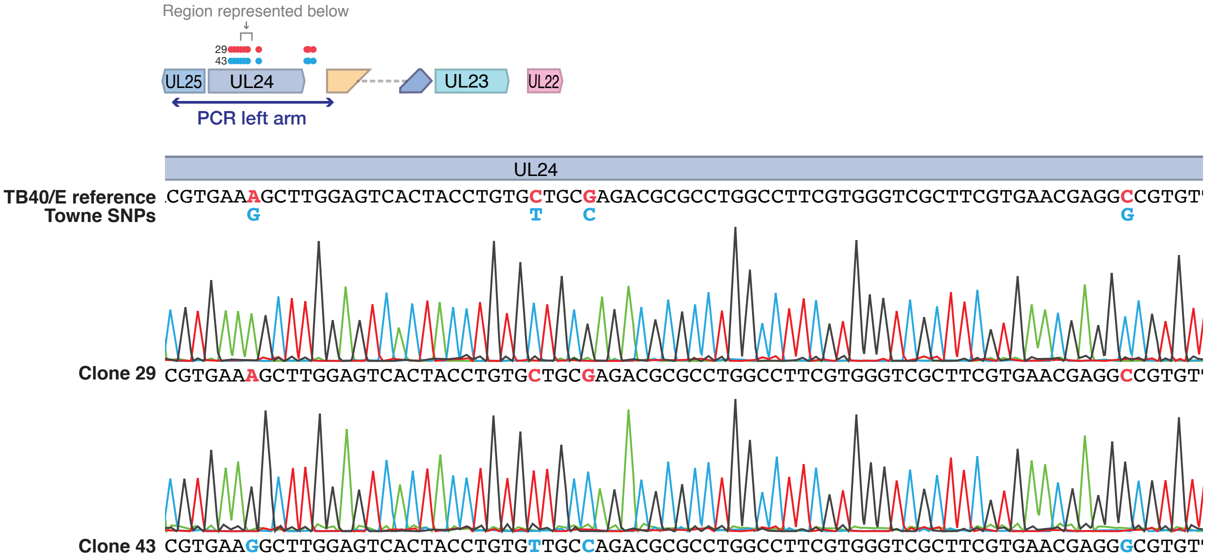

Supplementary Figure 3: Sanger sequencing of homology arms.
Example of Sanger sequencing of the left homology arm of two BAC clones. Clone 29 harbor Single Nucleotide Polymorphisms (SNPs) from TB40/E strain, and clone 43 has SNPs from Towne strain.

Run Statistics

	Biological Replicate 1		Biological Replicate 2
	Technical replicate 1	Technical replicate 2	
Active channels:	507	413	511
Mean read length:	8,786.7	5,397.6	610.2
Mean read quality:	8.8	6.3	7.1
Median read length:	1961	531	394
Median read quality:	9.6	7	7.5
Number of reads:	215,906	45,099	4,005,433
Read length N50:	41,608	51,314	729
Total bases:	1897092928	243427955	2444168486

Number and percentage of reads above quality cutoffs

>Q5:	195,035 (90.3%)	26,493 (58.7%)	3.315,315 (82.8%)
>Q7:	182,970 (84.7%)	22,550 (50.0%)	2,373,956 (59.3%)
>Q10:	80,464 (37.3%)	7,141 (15.8%)	164,324 (4.1%)
>Q12:	109 (0.1%)	20 (0.0%)	125 (0.0%)
>Q15:	0 (0.0%)	0 (0.0%)	0 (0.0%)
>Q6, >1kb	98,177 (37.6%)		271,924 (6.8%)
>Q6, >10kb	41,779 (16.0%)		9,926 (2.5%)
>Q6, >200kb	59 (0.0013%)		

Lengths of the 5 longest reads and their mean quality score

228,187 (Q7.9)	335,524 (Q8.7)	319,495 (Q9.2)
225,747 (Q8.8)	325,349 (Q7.9)	306,840 (Q7.4)
224,638 (Q7.7)	262,541 (Q7.7)	250,515 (Q8.3)
220,568 (Q7.8)	260,168 (Q9.0)	243,209 (Q8.8)
219,597 (Q7.8)	233,939 (Q10.0)	237,368 (Q8.3)

Mapping statistics

>Q6	mapped read (%)	175,389 (96.67%)	3,370,381(89.53%)
	on human hg38	71,753 (39.54%)	2,955,995 (78.52%)
	on hCMV-Towne	103,636 (57.12%)	414,386 (11.0%)
>Q6 >1kb	mapped read (%)	96,722 (98.52%)	269,868 (99.24%)
	on human hg38	13,776 (14.03%)	213,728 (78.59%)
	on hCMV-Towne	82,946 (84.4%)	56,140 (20.6%)
>Q6 >10kb	mapped read (%)	41,637 (99.66%)	9,883 (99.57%)
	on human hg38	328 (0.78%)	290 (2.92%)
	on hCMV-Towne	41,309 (98.9%)	9,593 (96.6%)

Histogram of read lengths

Biological replicate 1
Technical replicate 1

Biological replicate 1
Technical replicate 2

Biological replicate 2

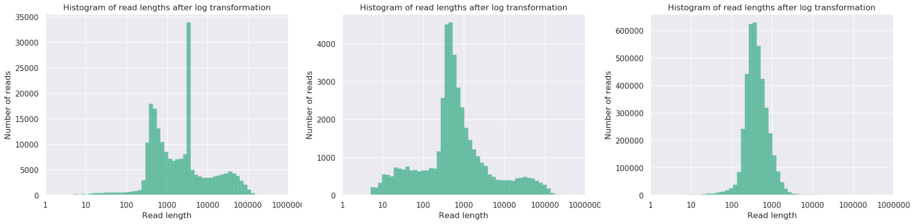

Supplementary Figure 4: Oxford Nanopore sequencing statistics.
Linear viral DNA was extracted from virions and subjected to long-read sequencing using Oxford Nanopore sequencing. The first biological replicate was sequenced in two technical replicates.

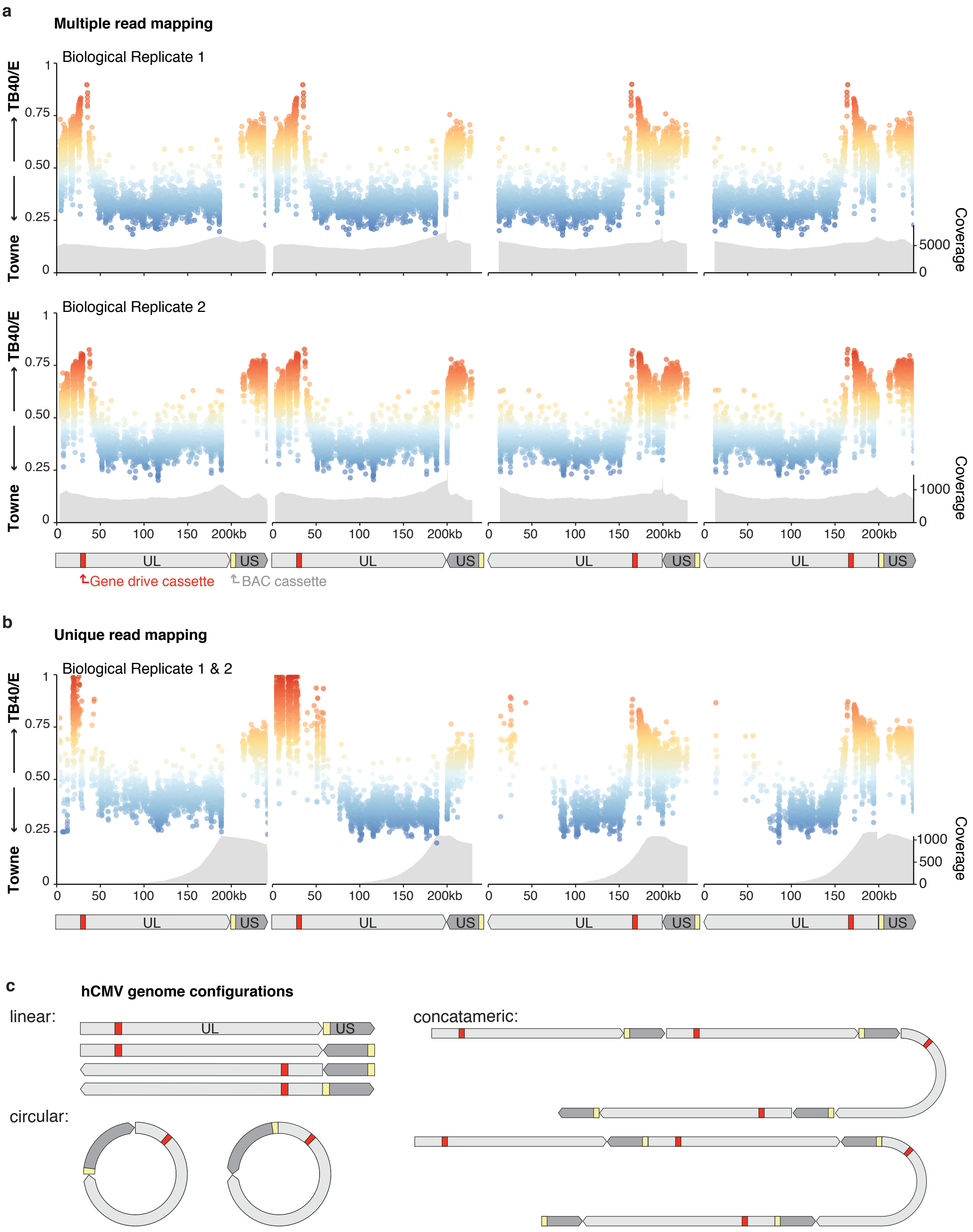

Supplementary Figure 5: SNP proportion in the four genome orientations.
a, Fraction of SNPs of Towne or TB40/E origin in the four different genome configurations. Each dot represents an individual SNP. Coverage gives the number of reads. Reads mapping ambiguously into one or multiple genome configurations. **b**, Same as a, but with reads mapping unambiguously into a unique genome configuration. **c**, The hCMV genome can be found in four linear or two circular/concatemeric genome configurations, depending on the respective orientation of the long (UL) and short (US) genome segments.

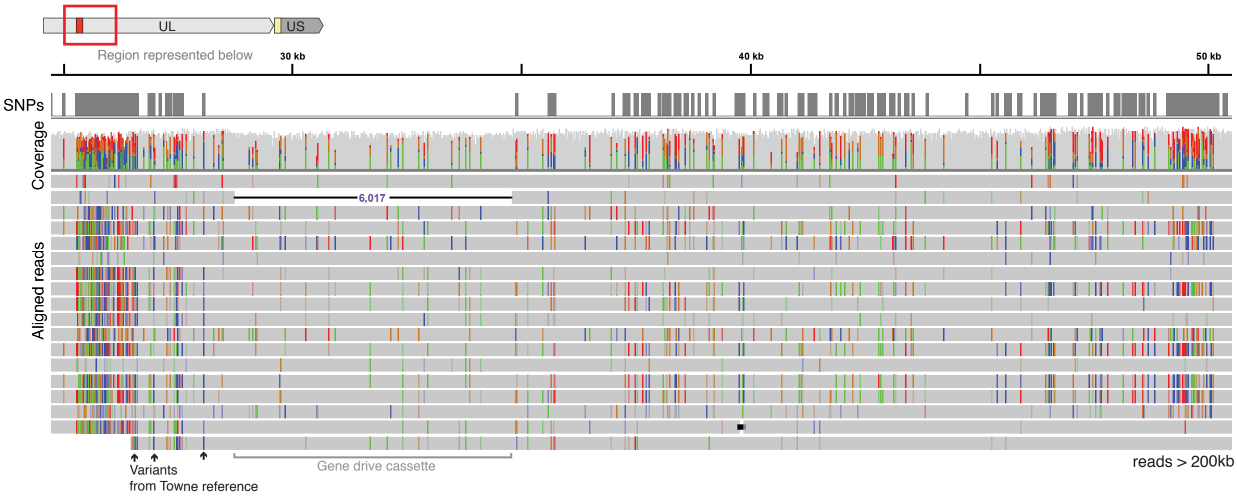

Supplementary Fig. 6: Reconstruction of recombination history from long reads.
IGV screenshot showing individual long reads (>200 kb) mapped into Towne reference sequence. Reads are noisy with numerous errors, but clusters of variants matching the SNP map allowed us to reconstruct the strain of origin.

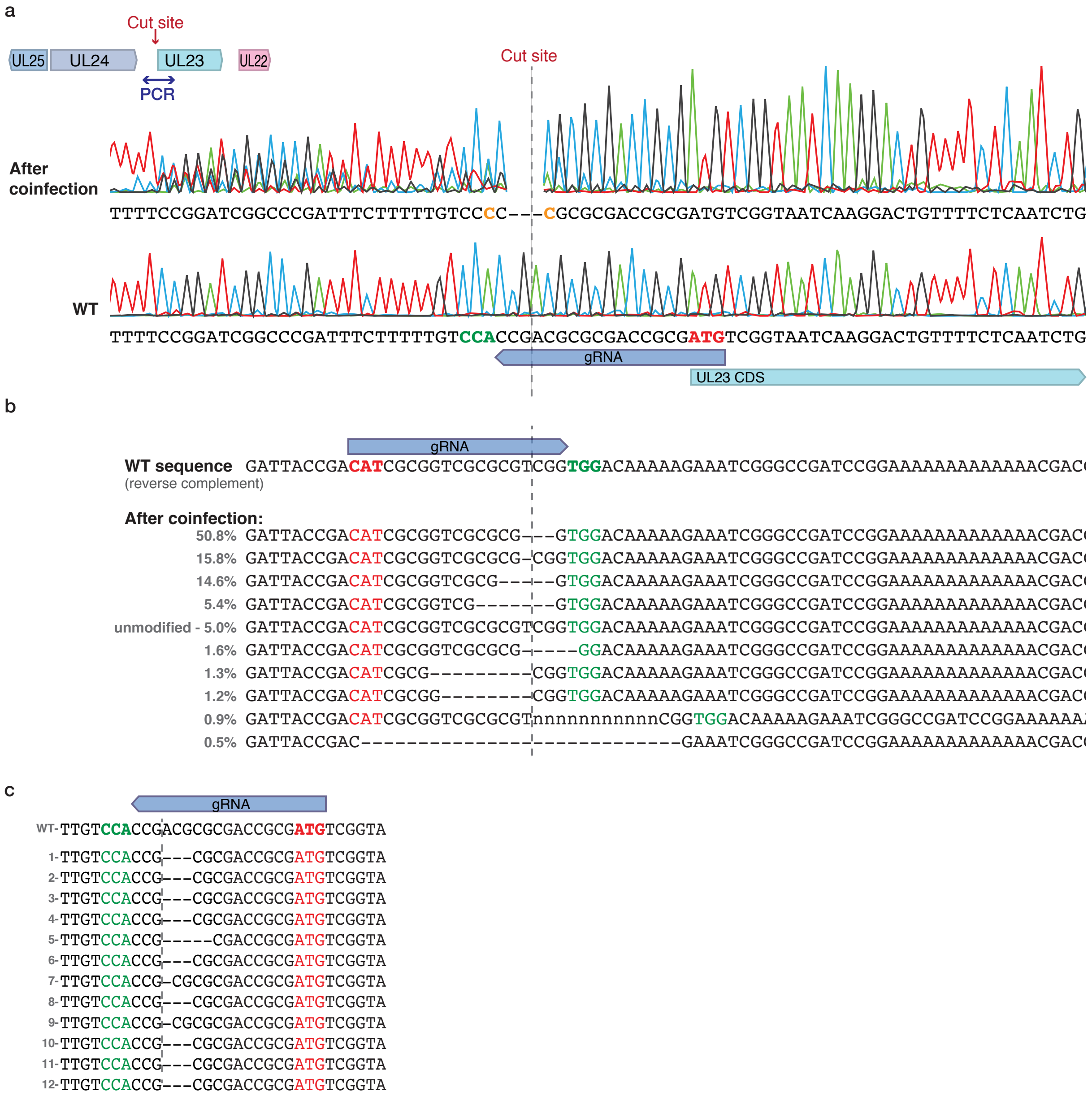

Extended Data Fig. 7: Sanger sequencing of drive-resistant viruses.
a, PCR and Sanger sequencing of the target site after coinfection with GD-mCherry and Towne-eGFP for 10 days. PCR was performed on the pool of coinfected cells. **b**, Deconvolution of sanger sequencing showing the relative contribution of each edits, using Synthego ICE online tool. **c**, Sequencing of 12 GFP-only viral clones isolated from 3 independent coinfection experiments.
Panel **a** and **b** show one example representative of multiple biological replicates, with a specific 3pb deletion representing 40-60% of edits.

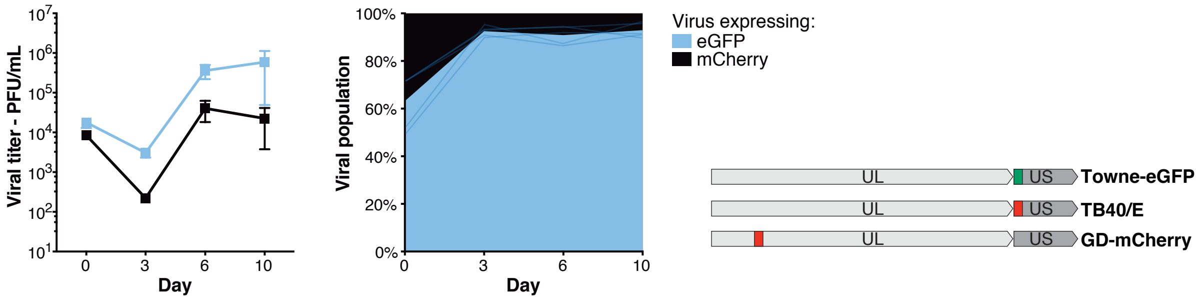

Supplementary Figure 8: Coinfection with Towne-eGFP and TB40/E.
Viral titer and proportion of viruses expressing eGFP alone, mCherry alone, after coinfection with equal amount of Towne-eGFP and TB40/E. n=5. Titers are expressed in PFU/mL. Error bars represent SEM between biological replicates. Source data are provided as a Source Data file.

a

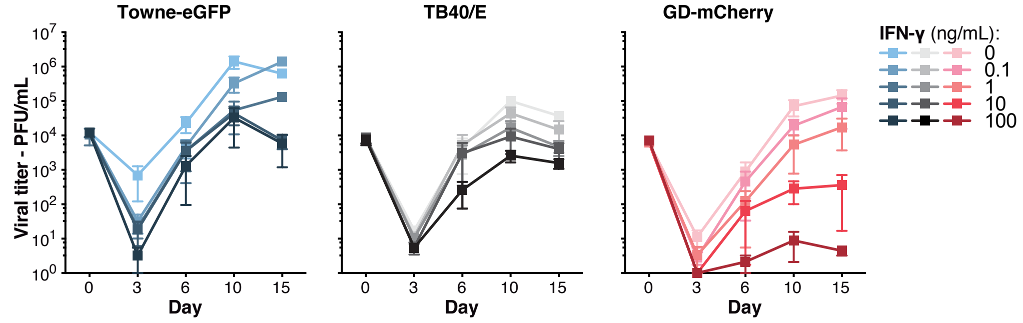

b

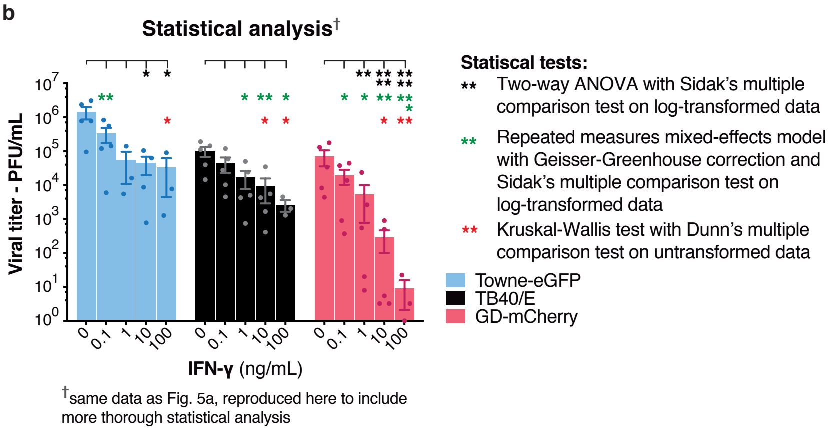

		Adjusted p value		
	IFN-γ	Anova	Repeated measure	Kruskal-Wallis
Towne-eGFP	0 vs. 0.1	0.4774	0.008	>0.9999
	0 vs. 1	0.0615	0.0841	0.1726
	0 vs. 10	0.0134	0.0864	0.0503
	0 vs. 100	0.0121	0.2044	0.0416
TB/40E	0 vs. 0.1	0.8671	0.0929	>0.9999
	0 vs. 1	0.2562	0.0319	0.1516
	0 vs. 10	0.0761	0.0068	0.0336
	0 vs. 100	0.0793	0.0189	0.0256
GD-mCherry	0 vs. 0.1	0.5753	0.0211	>0.9999
	0 vs. 1	0.0029	0.0182	0.2746
	0 vs. 10	<0.0001	0.0019	0.0164
	0 vs. 100	<0.0001	0.0009	0.0097

Supplementary Fig. 9: Viral titer in presence of IFN-γ.
a, Viral titer of single infections, in the presence of IFN-γ, measured by plaque assay. Data for D10 are also shown in Fig. 5a. and panel b. **b**, Viral titer at D10 in presence of increasing concentration of IFN-γ. Same figure as Fig. 4a with thorough statistical analysis. The table gives the adjusted p-value for every comparison for the different tests. Of note, two-way ANOVA or other parametric tests on untransformed data gave no significant results. Titers are expressed in PFU/mL. Error bars represent SEM between biological replicates. *, p-value < 0.05; **, p < 0.01; ***, p < 0.001; ****, p < 0.0001. n=3 (IFN-γ =100ng/mL) or n=5 (other concentrations). Source data are provided as a Source Data file.

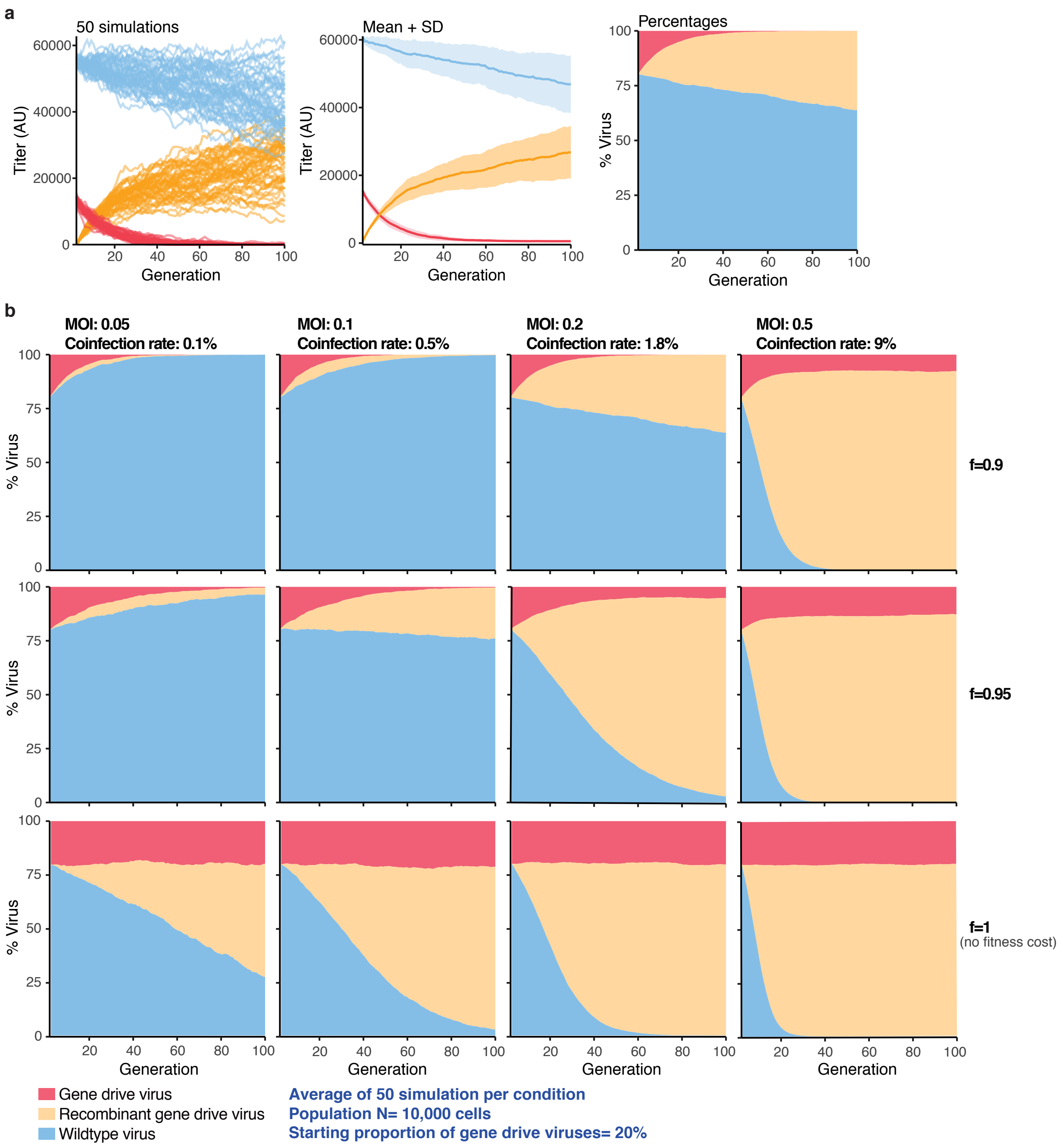

Supplementary Figure 10: Numerical simulation: variation of coinfection rate and fitness cost f of gene drive viruses.
Numerical simulation showing that the spread of gene drive virus in the wildtype population depends of the coinfection rate and of the replicative fitness cost f of gene-drive viruses. In these simulations, at each viral generation, N virtual cells were randomly infected and coinfected by N*MOI viruses, producing a new generation of viruses. When a cell is coinfected by wildtype (WT) and gene-drive viruses, WT viruses are converted to new gene-drive viruses. The coinfection rate is calculated from the MOI, assuming a Poisson distribution. **a**, example of a simulation with a replicative fitness cost f=0.9 for gene-drive viruses, and a coinfection rate of 1.8% (MOI=0.2). Left panel shows 50 independent simulations. Middle: mean and standard deviation of the 50 simulations. Right: mean proportion of the different viruses. **b**, Simulations with increasing coinfection rate and fitness cost of gene-drive viruses.
