# Supplementary material for: Viral gene drive in herpesviruses
Source: Nat Commun. 2020 Sep 28;11:4884. doi: 10.1038/s41467-020-18678-0 (PMC7522973; doi:10.1038/s41467-020-18678-0)
Supplement: Supplementary file 3 — Reporting Summary [file 41467_2020_18678_MOESM3_ESM.pdf]

nature research

Corresponding author(s): Marius Walter
Eric Verdin

Last updated by author(s): Jun 24, 2020

Reporting Summary

Nature Research wishes to improve the reproducibility of the work that we publish. This form provides structure for consistency and transparency in reporting. For further information on Nature Research policies, see our Editorial Policies and the Editorial Policy Checklist.

Statistics

For all statistical analyses, confirm that the following items are present in the figure legend, table legend, main text, or Methods section.

n/a	Confirmed	
☐	☒	The exact sample size (n) for each experimental group/condition, given as a discrete number and unit of measurement
☐	☒	A statement on whether measurements were taken from distinct samples or whether the same sample was measured repeatedly
☐	☒	The statistical test(s) used AND whether they are one- or two-sided<br>*Only common tests should be described solely by name; describe more complex techniques in the Methods section.*
☒	☐	A description of all covariates tested
☐	☒	A description of any assumptions or corrections, such as tests of normality and adjustment for multiple comparisons
☐	☒	A full description of the statistical parameters including central tendency (e.g. means) or other basic estimates (e.g. regression coefficient) AND variation (e.g. standard deviation) or associated estimates of uncertainty (e.g. confidence intervals)
☐	☒	For null hypothesis testing, the test statistic (e.g. F, t, r) with confidence intervals, effect sizes, degrees of freedom and P value noted<br>*Give P values as exact values whenever suitable.*
☒	☐	For Bayesian analysis, information on the choice of priors and Markov chain Monte Carlo settings
☒	☐	For hierarchical and complex designs, identification of the appropriate level for tests and full reporting of outcomes
☒	☐	Estimates of effect sizes (e.g. Cohen's d, Pearson's r), indicating how they were calculated

Our web collection on statistics for biologists contains articles on many of the points above.

Software and code

Policy information about availability of computer code

Data collection	Software used for data collection is described in the method section. It includes:<br>Nikon NIS Element AR 3.0 (imaging acquisition software), Graphpad Prism V8.1.1, Benchling.com
Data analysis	Code developed in the study for numerical simulations is available here: https://github.com/mariuswalter/ViralDrive.<br>Software used for the analysis are described in the method section of the paper. It includes:<br>Integrative Genome viewer (IGV v2.4.14), Albacore v2.3.3 (Oxford Nanopore), Nanoplot v1.0.0 (https://github.com/wdecoster/NanoPlot), Porechop v0.2.4 (https://github.com/rrwick/Porechop), nanofilt v2.5.0 ((https://github.com/wdecoster/nanofilt), nanolyse v1.0.0 (https://github.com/wdecoster/nanolyse), minimap2 v2.14 (https://github.com/lh3/minimap2), graphmap v0.3.0 (https://github.com/isovic/graphmap), nanopolish v0.10.2 (https://github.com/jts/nanopolish), ImageJ 2.1.0, samtools v1.10

For manuscripts utilizing custom algorithms or software that are central to the research but not yet described in published literature, software must be made available to editors and reviewers. We strongly encourage code deposition in a community repository (e.g. GitHub). See the Nature Research guidelines for submitting code & software for further information.

Data

Policy information about availability of data

All manuscripts must include a data availability statement. This statement should provide the following information, where applicable:

- Accession codes, unique identifiers, or web links for publicly available datasets
- A list of figures that have associated raw data
- A description of any restrictions on data availability

All sequencing data have been deposited in the Short Read Archive with BioProject accession no. PRJNA545115. Fig. 2e, 2f and Extended Data Fig.4-5-6 are associated with this data.

Code used in this manuscript for numerical simulation is available on GitHub (https://github.com/mariuswalter/ViralDrive), or described in the method section. Plasmids, viruses and other reagents developed in this study are available upon request and subject to standard material transfer agreements with the Buck Institute.

Field-specific reporting

Please select the one below that is the best fit for your research. If you are not sure, read the appropriate sections before making your selection.

- [x] Life sciences
- [ ] Behavioural & social sciences
- [ ] Ecological, evolutionary & environmental sciences

For a reference copy of the document with all sections, see nature.com/documents/nr-reporting-summary-flat.pdf

Life sciences study design

All studies must disclose on these points even when the disclosure is negative.

Sample size	No sample size calculation was performed. Sample size was large enough to determine statistically significant effects and followed the standard in the field.<br>Between 3 and 5 biological replicates were used for every experiment, as indicated in figure legends
Data exclusions	No data was excluded
Replication	Experiments were repeated independently at least 3-5 times, as indicated in the Figure legend. Every experiment described in the study was successfully reproducible,. Only the sequencing experiment was replicated twice.
Randomization	Randomization was not applicable in this study, samples were chosen based on the genotype of the viral strain.
Blinding	Investigators were not blinded to group allocation during experiments. It was not experimentally possible because sample collection and analyses were done by the same researcher. We treated and analyzed all samples in a similar procedure.

Reporting for specific materials, systems and methods

We require information from authors about some types of materials, experimental systems and methods used in many studies. Here, indicate whether each material, system or method listed is relevant to your study. If you are not sure if a list item applies to your research, read the appropriate section before selecting a response.

Materials & experimental systems

n/a	Involved in the study	
x		Antibodies
	x	Eukaryotic cell lines
x		Palaeontology and archaeology
x		Animals and other organisms
x		Human research participants
x		Clinical data
x		Dual use research of concern

Methods

n/a	Involved in the study	
x		ChIP-seq
x		Flow cytometry
x		MRI-based neuroimaging

Eukaryotic cell lines

Policy information about cell lines

Cell line source(s)	Human foreskin fibroblast cells were obtained from the ATTC (#SCRC-1041)
Authentication	Cells were directly received from the ATTC and no further authentication was performed
Mycoplasma contamination	Cells were regularly tested negative for mycoplasma by PCR
Commonly misidentified lines (See ICLAC register)	None
